# Genetic polymorphisms of *MMP1*, *MMP3 *and *MMP7 *gene promoter and risk of colorectal adenoma

**DOI:** 10.1186/1471-2407-6-270

**Published:** 2006-11-24

**Authors:** Astrid Lièvre, Jacqueline Milet, Jérôme Carayol, Delphine Le Corre, Chantal Milan, Alexandre Pariente, Bernard Nalet, Jacques Lafon, Jean Faivre, Claire Bonithon-Kopp, Sylviane Olschwang, Catherine Bonaiti-Pellié, Pierre Laurent-Puig, members of the ANGH group

**Affiliations:** 1 INSERM U775 et Université René Descartes, Paris, France; 2 INSERM, U535, Villejuif, France; 3 Université Paris-Sud, IFR69, Villejuif, France; 4 INSERM E106, Faculté de Médecine, Dijon, France; 5 Centre Hospitalier, Pau, France; 6 Centre Hospitalier, Montélimar, France; 7 Centre Hospitalier, Aix-en-Provence, France; 8 INSERM U599, Institut Paoli Calmettes, Marseille, France

## Abstract

**Background:**

Matrix metalloproteinases (MMP) have been shown to play a role in colorectal cancer (CRC). More recently, *MMP1*, *MMP3 *and *MMP7 *functional gene promoter polymorphisms have been found to be associated with CRC occurrence and prognosis. To document the role of MMP polymorphisms in the early step of colorectal carcinogenesis, we investigated their association with colorectal adenoma risk in a case-control study comprising 295 patients with large adenomas (LA), 302 patients with small adenomas (SA) and 568 polyp-free (PF) controls.

**Methods:**

Patients were genotyped using automated fragment analysis for *MMP1 *-1607 ins/del G and *MMP3 *-1612 ins/delA (*MMP3.1*) polymorphisms and allelic discrimination assay for *MMP3 *-709 A/G (*MMP3.2*) and *MMP7 *-181 A/G polymorphisms. Association between MMP genotypes and colorectal adenomas was first tested for each polymorphism separately and then for combined genotypes using the combination test. Adjustment on relevant variables and estimation of odds ratios were performed using unconditional logistic regression.

**Results:**

No association was observed between the polymorphisms and LA when compared to PF or SA. When comparing SA to PF controls, analysis revealed a significant association between *MMP3 *-1612 ins/delA polymorphism and SA with an increased risk associated with the 6A/6A genotype (OR = 1.67, 95%CI: 1.20–2.34). Using the combination test, the best association was found for *MMP3.1*-*MMP1 *(p = 0.001) with an OR of 1.88 (95%CI: 1.08–3.28) for the combined genotype 2G/2G-6A/6A estimated by logistic regression.

**Conclusion:**

These data show a relation between *MMP1 *-1607 ins/del G and *MMP3 *-1612 ins/delA combined polymorphisms and risk of SA, suggesting their potential role in the early steps of colorectal carcinogenesis.

## Background

Colorectal cancer (CRC) is one of the most common human malignancies with more than 300,000 cases both in the United States and in the European Union each year. The majority of the cases are sporadic and develop from a pre-malignant lesion, the adenomatous polyp [1]. Colonoscopic polypectomy has been proved to reduce significantly the incidence of colorectal cancer [2,3]. Therefore, the identification of factors associated with the development of colorectal adenoma represents a major goal in the prevention of colorectal cancer. Indeed, they could allow the selection of individuals at risk of CRC who may benefit from a screening by colonoscopy.

Matrix metalloproteinases (MMPs) are an important family of metal-dependant enzymes that are responsible for the degradation of extracellular matrix components. MMPs are involved in various physiologic processes, such as embryogenesis and tissue remodeling [4,5]. They also play a key role in invasion and metastasis of tumor cells which require proteolysis of basal membranes and extracellular matrix [6]. For a long time, MMPs were considered to be important almost exclusively in these two steps of carcinogenesis. However, recent studies suggested that MMPs are involved in several other processes associated with cancer development. Indeed, they regulate tumor growth and apoptosis, they promote angiogenesis, loss of cell adhesion, invasion and metastasis. Finally, some of them are also required in immune responses to cancer [7].

The role of MMPs in CRC has been described [8,9]. Numerous, including MMP1, MMP3 and MMP7 are overexpressed in colorectal tumors [10,11]. The expression of MMP7 was shown to correlate with Dukes' stage and increased metastatic potential [12,13] while MMP-1 expression was shown to be related to invasion, metastasis and prognosis [14-16]. Moran *et al*. demonstrated that MMP3 expression was significantly lower in CRC with high microsatellite instability which are known to have a better clinical outcome than CRC without microsatellite instability [17]. This observation suggests that MMP3 could be implicated in tumor invasion, lymph node involvement and metastatic spread in CRC. MMPs are overexpressed in a variety of premalignant tumor tissues, including colorectal adenoma [18-20] and MMP7 has been shown to be important in the growth of early colonic adenomas and their transformation into invasive cancer [21].

A functional single nucleotide polymorphism (SNP) has been reported in the *MMP1 *gene promoter that consists in a guanosine (G) insertion at position -1607. This SNP generates a new 5'-GGA-3' core recognition sequence for members of the Ets family of transcription factors [22]. *In vitro*, the homozygous 2G/2G genotype results in an increased transcription activity compared to the 1G/1G genotype. *In vivo*, an association was found between the 2G allele and MMP1 overexpression in ovarian cancer tissue [23]. The *MMP1 *2G/2G genotype was reported to be linked to an increased risk of CRC [24,25]. Indeed, the 2G allele was shown to favor invasion, metastasis and prognosis [25,26]. A SNP corresponding to an insertion/deletion of an adenosine (A) at position -1612 of the *MMP3 *gene promoter was also described and shown to interfere with transcriptional activity [10]. In a case-control study, Hinoda *et al*. found that the frequency of the 6A allele was significantly higher in CRC patients than in controls, as the frequency of the 6A/6A genotype compared to the 5A/6A + 5A/5A genotypes [24]. In this study, *MMP1 *and *MMP3 *were in significant linkage desequilibrum and the most frequent haplotype 2G-6A was significantly increased in CRC patients compared to controls. Concerning the other *MMP3 *polymorphisms, four are in substantial linkage desequilibrum with the -1612 ins/del A (-1986 T/C, -1346 A/C, -376 G/C, and +802 A/G), except one (-709 A/G) which is not of proven functional importance [27]. In the MMP7 gene promoter region, two SNPs (-181 A/G and -153 C/T) have been shown to modify the gene transcription activity [28]. Recently, both SNPs have been associated with CRC susceptibility and the -181 G/G genotype was linked to distant metastasis [29]. These findings are in favor of the role of *MMP1*, *MMP3 *and *MMP7 *functional gene polymorphisms in colorectal carcinogenesis. They are localized in a MMP cluster of 400 kb at 11q21–23 that counts 9 MMPs (MMP-1, 3, 7, 8, 10, 12, 13, 20, 27).

To explore whether *MMP1*, *MMP3 *and *MMP7 *gene promoter polymorphisms are involved in the early step of colorectal carcinogenesis, we investigated the relation between these polymorphisms and the risk of colorectal adenoma in a case-control study.

## Methods

### Selection of cases and controls

The GEADE study is a case-control and family study of patients with high-risk adenomas (larger than 9 mm). The data were obtained from 18 participating gastroenterology units of general hospitals in France (table 3). From September 1995 to March 2000, 306 consecutive patients with newly diagnosed colorectal large adenoma (LA) were enrolled in the study. Subjects with history of cancer, familial adenomatous polyposis, established hereditary non-polyposis colorectal cancer and inflammatory bowel disease had been excluded. In order to be able to distinguish genetic factors involved in the occurrence of adenomas or in their growth, 307 cases with small adenomas (SA) (smaller than 0.5 cm) and 572 polyp-free (PF) controls (with normal colonoscopy) were enrolled in the same units. All patients and controls were Caucasian. Information on indication of colonoscopy, family history of CRC, and completeness of colonoscopy was also collected for all patients and controls. Controls (2 per case) were matched to LA cases by age, gender, and geographic area (five regions: Paris area, North-East, Centre, South-West and South-East, France) (table 3). Patients with SA were relatively rare and could not be matched with LA cases. Blood specimens were obtained at the time of the colonoscopy and were kept only if adenoma was diagnosed after histological examination (for LA and SA cases). Twenty individuals could not be genotyped because biological material was not sufficient: 11 cases with LA, 5 cases with SA and 4 PF controls. The final sample was thus: 295 cases with LA, 302 cases with SA and 568 PF controls. A complete description of the patients sample is given in Cottet *et al*. [30]. All patients and controls gave their informed consent and the study was approved by an ethics committee for biomedical research (Le Kremlin-Bicêtre) and by the National Committee CNIL (Commission nationale de l'informatique et des libertés).

### MMP1, MMP3 and MMP7 genotyping

The *MMP1 *-1607 ins/del G, *MMP3 *-1612 ins/del A, *MMP3 *-709 A/G and *MMP7 *-181 A/G polymorphisms were named respectively *MMP1*, *MMP3.1*, *MMP3.2 *and *MMP7 *polymorphisms.*MMP1 *and *MMP3.1 *have been studied by genotyping lymphocyte DNA by multiplex PCR-based approach using the following primers: 5' [6FAM]-GCCCTCTTGAACTCACATGTTATG-3' and 5'-ACTTTCCTCCCCTTATGGATTCC-3' for *MMP1*, 5'-GTCCTCATATCAATGTGGCCAAA-3' and 5' [6FAM]-CGGCACCTGGCCTAAAGAC-3' for *MMP3.1 *polymorphism as previously described [31]. After amplification and dilution, fragments were separated on a ABI Prism 3700 DNA Genetic Analyzer (Applied Biosystems).

The *MMP3.2 *and *MMP7 *polymorphisms were studied by real-time PCR allelic discrimination assay on ABI 7900 HT Sequence Detection System (Applied Biosystems) as previously described [31]. For the *MMP7 *DNA amplification was performed with forward 5'-AGTCAATTTATGCAGCAGACAGAAA-3' and reverse 5'-GTGTTATTTTTCATTAACTAAAACGAGGA-3' primers and allelic discrimination was performed by the use of specific probes for each allele respectively labelled with fluorescent reporter dyes VIC and FAM: 5'ACAATGTATTTGTCTTTC-3' and 5'-CAATGTATTCGTCTTTC-3'. For the *MMP3.2 *polymorphism, a mix containing specific primers and fluorescent probes designed by Applied Biosystems (Taqman SNP Genotyping Assay, ID C_3047714_10) was used. Some QC blinded samples were distributed throughout the runs and genotyped for concordance of results and control samples without DNA samples were systematically included in each MMP genotyping plate.

### Statistical analysis

Hardy-Weinberg proportions were tested for each polymorphism. Linkage disequilibrium (LD) between pairwise loci was estimated using the measure D' [32].

Association was first tested for each polymorphism separately. For each polymorphism, genotype-specific odds ratio (OR) and 95% confidence intervals (CI) were computed using unconditional logistic regression adjusted on matching factors and Wald test was used to assess the global effect of this SNP. The homozygous genotype for the more frequent allele among controls was set as the reference class. Homogeneity of allele frequencies within regions was previously tested. Tests of homogeneity and unconditional logistic regression were done using STATA.

The association was further examined using the combination test, a method developed by Jannot *et al*. [33] that allows for the analysis of all possible combinations of 1 to n tightly linked SNPs in order to test their association with the disease. For each SNP combination, the method computes a statistic test contrasting the genotypic (or haplotypic) distribution between cases and controls. Because all these tests are correlated (many of them are nested in each others, and the SNPs are likely to be in LD), a permutation procedure is implemented which displays a significance level adequately adjusted for multiple testing. In a second step, among the significantly associated combinations, the most parsimonious one can be identified by comparing nested combinations using chi2 tests.

With the 4 SNPs of the present study, a total of 15 SNP combination were tested (4 single SNP tests, 6 two-SNP tests, 4 three-SNP tests and 1 four-SNP test).

First, we used the FAMHAP12 software to apply this method by performing haplotypic tests [34]. Then, the COMBINTEST (Jannot, personal communication) was used to perform genotypic tests.

As the combination test does not yet allow an adjustment on confounders, when association was found significant, the combination was further tested using unconditional logistic regression and the risks associated with the different genotypes were estimated.

## Results

Table 1 shows some characteristics of the cases and controls. LA cases were slightly older than SA and PF controls, but there was no age difference between LA and SA patients, despite the absence of matching. The sex ratio (male/female) was similar in LA and PF because of matching, and slightly higher in LA than in SA patients.

The distribution of genotypes for the 4 polymorphisms was consistent with Hardy-Weinberg equilibrium (p = 0.65 for *MMP1*, p = 0.13 for *MMP3.1*, p = 0.36 for *MMP3.2 *and p = 0.10 for *MMP7*). As shown in table 2, three of the four polymorphisms (*MMP1*, *MMP3.1 *and *MMP3.2*) were in LD but *MMP7 *was only in slight LD with *MMP3.2 *polymorphism (D' = 0.18) and was not found in LD with the other ones (D'< 0.05).

Some differences in allele frequencies were found between centres inside the regions defined for matching (the homogeneity of allele frequencies was rejected for 5 tests among the 20 ones performed). Although such differences might be due to chance, there might exist some kind of stratification within the 5 regions initially defined, as found for other polymorphisms [35]. Therefore, we adjusted analyses on smaller groups of centres to protect against spurious association due to possible remaining population admixture. Centres with too few participants were pooled with the geographically closest ones, which led to eleven categories (table 3).

Results of analyses by single polymorphisms for LA vs PF, LA vs SA and SA vs PF are presented in table 4. No association was observed between the polymorphisms and LA when compared to PF or SA. Conversely, a significant effect of *MMP3.1 *polymorphism was found when comparing SA to PF controls (p = 0.008, Wald test). The data suggest an opposite effect of 6A allele depending on whether one or two copies were present. Compared with the 5A/5A genotype, the 6A/6A variant genotype appeared associated with an increased risk of SA whereas 5A/6A was inversely associated but the ORs were not significantly different from 1.0. When we considered a recessive model, the genotype 6A/6A was this time clearly associated with a higher risk of SA compared with the genotypes 5A/5A and 5A/6A pooled together (OR = 1.67, 95%CI: 1.20–2.34).

The haplotypic analysis with the combination test did not reveal any effect of the 4 polymorphisms on LA. When comparing SA vs PF, the test was globally significant after correction for multiple testing (p = 0.012). The combination of polymorphisms found associated with SA with the lowest p-value was *MMP1-MMP3.1 *(p = 0.001, uncorrected). This was the best combination as no other combinations of 1 or 2 SNP was significantly associated with SA (in particular, when *MMP3.1 *was considered alone, the p-value was 0.059). The genotypic analysis by the combination test provided quite similar results with a p-value of 0.03 after correction for multiple testing. The lowest p-value was found for the same combination *MMP1-MMP3.1 *(p = 0.003, uncorrected). The only difference was that *MMP3.1 *SNP alone was significantly associated with SA (p = 0.011). As in the haplotypic analysis, the comparison between statistics of MMP1-MMP3.1 and MMP3.1 combinations showed that the combination MMP3.1-MMP1 was the most significantly associated one.

An association between combined *MMP1-MMP3.1 *genotypes and SA was confirmed by logistic regression adjusted on centre (p = 0.002) (table 5). The same opposite effect of 5A and 6A alleles shown in the SNP by SNP analyses was found for *MMP3.1 *but only for the carriers of genotype 2G/2G at the *MMP1 *locus.

## Discussion

We investigated the role of *MMP1*, *MMP3 *and *MMP7 *promoter polymorphisms as colorectal cancer risk factors in a case-control study of cases with large adenomatous polyps (n = 295), versus small adenomatous polyps cases (n = 302) or PF controls (n = 568). These polymorphisms are known to modulate transcriptional activity, except for one (*MMP3 *-709 A/G) that is not of proven functional importance [27].

The distribution of genotypes for the four polymorphisms was consistent with Hardy-Weinberg equilibrium. *MMP1*, *MMP3.1 *and *MMP3.2 *polymorphisms were found in LD, as previously in several studies, the *MMP1 *2G and *MMP3.1 *6A alleles as well as the *MMP1 *1G and *MMP3.1 *5A alleles were in LD with a D' coefficient between 0.50 and 0.92 according to the ethnic origin of the patients [24,26,31]. Concerning the *MMP3.2 *-709 A/G polymorphism, few data are available but one study reported a substantial LD between five *MMP3 *promoter polymorphisms, except for this one, which had a lower frequency of the minor allele (0.2 vs > 0.4 for the minor allele of the other polymorphisms) [27]. The *MMP7 *polymorphism was not found in LD with the others (D' < 0.05), which is consistent with the literature [31].

In this case-control study, the *MMP3.1 *6A/6A genotype was significantly associated with an increased risk of SA when compared to PF controls (OR = 1.67, 95%CI: 1.20–2.34, under a recessive model) while no association was observed between the MMP polymorphisms analyzed and LA when compared to PF or SA. Moreover, the combination test revealed a stronger effect of the *MMP1-MMP3.1 *2G/2G-6A/6A combined genotype. These results suggest that *MMP1 *polymorphism, which was not found to influence adenoma risk when taken alone, may have a role by interacting with the effect of *MMP3.1*. This finding underlines the importance of using the combination test for demonstrating combined effects of polymorphisms with little or no individual effect. Indeed, this test has been shown to be particularly powerful to detect the effect of polymorphisms when several polymorphisms interact with low marginal effect of each SNP and when one of the SNPs masks the expression of the other ones [33]

Our data suggest that the *MMP3.1 *polymorphism, and especially the *MMP1-MMP3.1 *2G/2G-6A/6A combined genotype might play a role at the earliest step of colorectal carcinogenesis by promoting the development of adenomas from normal colon epithelial cells. However, the following steps of colorectal carcinogenesis, in particular adenoma growth, could require the contribution of other factors. These factors could mask the effect of *MMP1-MMP3.1 *combined genotype and explain the absence of association when LA patients are compared to PF controls. However, these preliminary results must be taken with caution and have to be confirmed.

The *MMP3.1 *6A/6A genotype has already linked to the development of CRC. Indeed, its frequency was significantly higher in CRC patients than in controls when compared to the 5A/5A + 5A/6A genotypes in a case-control study [24]. These results are inconsistent with the hypothesis that the increased transcriptional level of *MMP3 *increases CRC susceptibility as the 6A/6A genotype is associated with the lowest transcriptional level [36]. One explanation may be that *MMP3 *is indirectly involved in the development of CRC [24]. The presence of an increased risk of adenomatous polyps of the colon in coronary atherosclerosis patients [37,38] suggested that the association of the MMP3 6A/6A genotype and CRC is due to its link with an increased atherosclerosis risk [39-41]. These speculations should obviously be taken with caution and the mechanisms underlying the potential involvement of *MMP3.1 *polymorphism in colorectal carcinogenesis remain to be clearly determined.

Different studies showed that some MMP, including MMP2, MMP7 and MMP9 are expressed in colorectal adenomas that are well established premalignant lesions of colorectal cancers [18-20,42], implying their role other than extracellular matrix destruction and metastasis in cancer development and progression. The expression of E1AF, an Ets family transcriptional factor that plays a role in the progression of CRC, has been shown to be associated with the expression of MMP1 and MMP7 in CRC tissues [43,44]. Moreover, E1AF has been reported to be a potent activator of cyclooxygenase-2 (COX-2) transcription [45] and it is established that COX-2 plays an important role in the early stages of colorectal carcinogenesis [46]. Finally, absence of MMP7 may result in a significant reduction in mean tumour number and average tumour diameter in Min (multiple intestinal neoplasia) mice deficient in this MMP [21]. These findings suggest that some MMP might contribute to early tumour development, especially in tumours of gastrointestinal tract. Although several studies analyzed the expression of different MMP in colorectal adenomas, none had investigated the role of functional MMP polymorphisms in the development of these premalignant lesions despite evidence of their implication in colorectal carcinogenesis.

In the present study, the *MMP3.1 *6A/6A genotype was significantly associated with an increased risk of SA. Moreover, the analysis of the effects of combined MMP genotypes by the means of the combination test allowed to find an indirect role of *MMP1 *polymorphism in this early step of colorectal carcinogenesis since a potentialisation of the effect of *MMP3.1 *polymorphism by this polymorphism has been shown. Such an effect would not have been identified if a separated analysis of each MMP genotype had been performed, underlining the relevance of the combination test.

## Conclusion

These data show a relation between *MMP1 *-1607 ins/delG and *MMP3 *-1612 ins/delA combined polymorphisms and risk of colorectal SA, suggesting their potential role in the early steps of colorectal carcinogenesis.

## Abbreviations

CRC; colorectal cancer

LA; large adenoma

MMP; matrix metalloproteinases

PF; polyp free

SA; small adenoma

SNP; single nucleotide polymorphism

## Competing interests

The author(s) declare that they have no competing interests.

## Authors' contributions

AL and DLC carried out the genotyping under the supervision of PLP. AP, BN, JL and the ANGH group participated to the inclusion of patients in the study and performed colonoscopies with polypectomy. SO carried out the DNA extraction. JM, JC and CBP performed the statistical analysis. CBP, CM, JF, CBK, SO, AP and JL participated in the design of the study and/or the management of the data. AL, CBP, JM and PLP drafted the manuscript. All authors read and approved the final manuscript.

## Pre-publication history

The pre-publication history for this paper can be accessed here:



## Figures and Tables

**Table 1:** Characteristics of the study population

	Large adenomas	Small adenomas	Polyp free controls
N	295	302	568
Age, mean (SD)	62.3 (13.1)	60.6 (12.1)	60.7 (12.4)
Sex ratio (male/female)	1.68	1.24	1.52

SD; standard deviation

**Table 2:** Linkage disequilibrium (D') between pairwise polymorphisms

Polymorphisms D' (p-value)	*MMP3.1*	*MMP7*	*MMP3.2*
*MMP1*	0.429 (< 0.001)	0.016 (0.686)	0.761 (< 0.001)
*MMP3.1*		0.040 (0.231)	0.946 (< 0.001)
*MMP7*			0.185 (0.005)

**Table 3:** Distribution of cases and controls in the different centres and groups of centres

Region	Centre	Number of individuals by centre		Number of individuals by group of centres	
		
		All cases	Controls	All cases	Controls
Paris area	Le Chesnay	10	15		
	Meaux	45	32	55	47
	Orleans	16	7		
	Le Mans	49	49	65	56
	Dreux	12	39	12	39

North-East	Evreux	25	16		
	Beauvais	8	19		
	Soissons	1	1		
	Compiègne	24	16	58	51
	Metz	45	49	45	49

Centre	Vichy	38	2		
	Bourg en Bresse	76	51	114	53
	Beaune	37	37	37	37

South-West	Tarbes	31	29	31	29
	Pau	67	54	67	54

South-East	Toulon	10	10		
	Aix en Provence	58	61	68	71
	Montélimar	45	36	45	36

**Table 4:** Association between genotypes of MMP and colorectal adenomas

	Large adenoma patients (LA)	Polyp free controls (PF)	Small adenoma patients (SA)	LA vs PF		LA vs SA		SA vs PF	
				
				OR* (95%CI)	P**	OR* (95%CI)	P**	OR* (95%CI)	P**
***MMP1***									
1G/1G	81	154	81	1		1		1	
1G/2G	139	285	139	0.94 (0.66–1.33)		1.04 (0.69–1.56)		0.96 (0.67–1.37)	
2G/2G	72	122	79	1.18 (0.78–1.78)	0.491	0.90 (0.56–1.43)	0.792	1.22 (0.81–1.84)	0.431

***MMP3.1***									
5A/5A	70	130	68	1		1		1	
5A/6A	139	291	132	0.87 (0.60–1.25)		1.10 (0.72–1.69)		0.85 (0.58–1.24)	
6A/6A	73	126	93	1.11 (0.73–1.69)	0.391	0.78 (0.48–1.25)	0.250	1.50 (0.99–2.28)	0.008

***MMP3.2***									
AA	182	358	194	1		1		1	
AG	105	187	92	1.10 (0.81–1.50)		1.20 (0.83–1.73)		0.87 (0.64–1.20)	
GG	7	19	16	0.72 (0.29–1.78)	0.615	0.49 (0.19–1.27)	0.175	1.34 (0.65–2.75)	0.464

***MMP7***									
AA	92	187	99	1		1		1	
AG	138	259	136	1.08 (0.77–1.50)		1.06 (0.72–1.56)		0.98 (0.70–1.38)	
GG	64	119	67	1.17 (0.78–1.77)	0.742	1.08 (0.67–1.72)	0.944	1.14 (0.76–1.71)	0.746

* OR adjusted on age, sex, and group of centres

** P: p-value for the Wald test assessing the global effect of SNP, uncorrected for multiple testing

**Table 5:** Combined effect of *MMP1 *and *MMP3.1 *loci in small adenomas

Genotypes		Polyp free (n)	Small adenoma (n)	OR* (95% CI)
*MMP1*	*MMP3.1*			
1G-1G	5A-5A	76	47	1
1G-1G	5A-6A	58	26	0.77 (0.41–1.43)
1G-1G	6A-6A	19	5	0.37 (0.12–1.12)
1G-2G	5A-5A	46	18	0.68 (0.34–1.38)
1G-2G	5A-6A	174	88	0.83 (0.52–1.33)
1G-2G	6A-6A	56	33	1.13 (0.62–2.04)
2G-2G	5A-5A	8	3	0.63 (0.15–2.62)
2G-2G	5A-6A	59	18	0.45 (0.23–0.89)
2G-2G	6A-6A	51	55	1.88 (1.08–3.28)

* OR adjusted on age, sex and group of centres
